# Novel fetal phenotype of TAF8 deficiency

**DOI:** 10.1038/s41431-024-01679-8

**Published:** 2024-08-21

**Authors:** Golan Nadav, Marwan Odeh, Aviv Mesika, Yael Abarbanel Har-Tal, Moshe Goldfeld, Tania Zalatkin, Alejandro Livoff, Raghad Jeris Khoury, Inshirah Sgayer, Liat Ben-Sira, Limor Kalfon, Tzipora C. Falik-Zaccai

**Affiliations:** 1 Institute of Human Genetics, Galilee Medical Center, Nahariya, Israel; 2 The Unit for Ob/Gyn Ultrasound, Galilee Medical Center, Nahariya, Israel; 3 Azrieli Faculty of Medicine, Bar Ilan University, Safed, Israel; 4 MRI Imaging Unit, Galilee Medical Center, Nahariya, Israel; 5 Department of Pathology, Galilee Medical Center, Nahariya, Israel; 6 Department of Radiology, Division of Pediatric Radiology, Dana Children’s Hospital, Tel Aviv Sourasky Medical Center and Faculty of Medicine, Tel Aviv University, Tel Aviv, Israel

**Keywords:** Clinical epigenetics, Clinical genetics

## Abstract

TAF8 is part of the transcription factor TFIID complex. TFIID is crucial for recruiting the transcription factor complex containing RNA polymerase II. TAF8 deficiency was recently reported as causing a severe neurodevelopmental disorder in eight patients. We have ascertained three Muslim Arab couples with fetal brain malformations. Clinical, imaging, pathological, biochemical, and molecular analyses were performed. Pre-natal ultrasound performed in four pregnancies revealed massive cerebellar atrophy, microcephaly, cerebral and corpus callosum (CC) anomalies. Pre-natal MRI studies of two of the affected fetuses confirmed microcephaly, small vermis, abnormal sulcation pattern with malformation, and shortening of CC. The fetuses were found to carry a novel likely pathogenic homozygous variant (c.45 + 5 G > A) of *TAF8*, predicted to affect splicing and presenting autosomal recessive inheritance. Post-mortem examinations confirmed the imaging studies in one fetus. Dysmorphic features including hypertelorism, wide nasal bridge, clinodactyly, and hirsutism were present. Western blotting analysis in fibroblasts of an affected fetus demonstrated a significant reduction of TAF8 protein. We determined high expression levels of TAF8 which progressively diminish in fetal brains of WT mice. We report for the first time the fetal presentation of TAF8 deficiency due to a novel genetic variant, and study TAF8 presence during fetal and neonatal periods in mouse brains. Our study may contribute to understanding the role of TAF8 in the developing human brain.

## Introduction

TATA-box binding protein-associated factor 8 (TAF8) is a member of TATA-binding protein (TBP)-associated factors (TAFs) group [1] which binds with TBP to the TATA-box sequence in the promoter region [2] as part of the transcription factor IID (TFIID) complex. TFIID is essential for RNA polymerase II (pol II) function during basal transcription machinery in eukaryotes [3]. Pol II has a critical role in gene expression during different stages of embryonic development [4] and studies have shown that defects in its function lead to various diseases and developmental disorders [5]. Furthermore, the literature describes several pathogenic genetic variants in *TAF* genes including *TAF1*, *TAF2*, *TAF6*, and *TAF13* [6]. The main clinical manifestations of patients with *TAF*-related disorders are global developmental delay and dysmorphic features [7–9]. Recently, Wong et al. reported pathogenic genetic variants in *TAF8* that have been associated with severe psychomotor retardation, feeding problems, microcephaly, growth retardation, spasticity, and epilepsy [1]. TAF8 is also involved in regulating the activity of TFIID and has a role in the recruitment of other proteins to the complex [10]. Furthermore, TAF8 is a key player in the regulation of gene expression [2] and the stabilization of the TFIID complex [3]. There is, however, no evidence that links the expression of TAF8 and normal brain formation. We report here for the first time the pre-natal presentation of four fetuses with a novel homozygous pathogenic genetic variant of *TAF8* leading to the elimination of the expression of TAF8 in the affected fetal brain and determining the expression of TAF8 in the developing brains of mice.

## Methods

The guardians of the affected individuals signed an informed consent form for participation in this study. The Israeli Supreme Helsinki Committee approved the study; (GMC-991110, NHR-58-17).

**Fig. 1:**
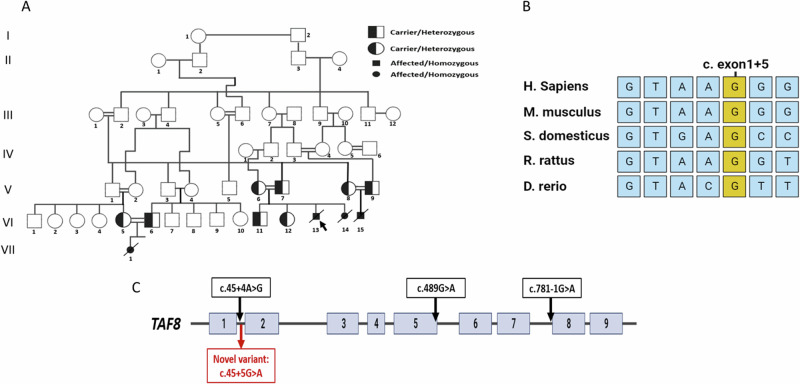
Causative genetic variant in *TAF8*. **A** Pedigree of the extended Muslim family presenting a novel pathogenic genetic variant in *TAF8*. **B** Splicing sequence alignment in *TAF8* orthologs shows high conservation of the c.45 + 5 G > A variant (highlighted in yellow) and almost complete conservation of the nearby residues (highlighted in light blue). **C** Schematic representation of *TAF8* gene (bars = exons) and previously reported pathogenic variants including the novel variant c.45 + 5 G > A reported here for the first time.

Pregnant woman #1 (V6, Fig. 1A), a 22-year old, healthy and in a consanguineous marriage (first cousins once removed) who, in her third pregnancy (VI13, Fig. 1A) presented for fetal neuro-sonography exam since earlier fetal scan at 23 + 1 weeks’ gestation (WG) had revealed microcephaly and hypoplasia of corpus callosum (CC). Later, in the 19th week of her fourth pregnancy (VI14, Fig. 1A), she presented with similar fetal brain abnormalities in fetal anatomy. In her fifth pregnancy, she had CVS testing at 11 WG.

Pregnant woman #2 (V8, Fig. 1A), a 24-year-old in her first pregnancy from a consanguineous marriage (first cousins), presented to us after her fetal scan at 23w + 2d revealed a short CC, cerebellar hypoplasia, and microcephaly (VI15, Fig. 1A).

Pregnant woman #3 (VI5, Fig. 1A), a 22-year-old in her first pregnancy from a consanguineous marriage (first and second cousins), presented at 23 WG with fetal anatomy scan that revealed delayed fetal growth (equivalent to 19–20 weeks), microcephaly, and severe brain atrophy (VII1, Fig. 1A). An earlier fetal scan at 15 WG reported bilateral cervical cysts with no additional abnormal findings. All three women are family-related (Fig. 1A).

### Imaging studies

#### Ultrasound studies

Fetal neuro-sonography exams were performed using a GE Voluson E10 machine, and fetal CNS assessment involved axial, sagittal, and coronal views, utilizing both abdominal and transvaginal approaches.

#### Brain MRI

Pregnant women #1 (V6, Fig. 1A) and #2 (V8, Fig. 1A) underwent fetal brain MRI (3T Siemens Skyra MRI scanner or GE, discovery MR 450 scanner) at 28 and 29 WG, respectively. Following a localizing gradient-echo sequence, ultra T2-weighted single-shot fast-spin echo MR images were performed in the axial, coronal, and sagittal planes.

Pregnant woman #3 (VI5, Fig. 1A) underwent termination of pregnancy at 20 WG, post-mortem MRI was acquired immediately after the procedure using 1.5T (GE, OPTIMA 450 W), T1- and T2-weighted, FSE, 3D cube, SWI, DWI sequences.

### Pathological analysis

A female fetus (VI14 Fig. 1A) with growth parameters consistent with 20 WG arrived for fetal PM studies including whole body and brain using standard protocol [11].

#### Cell culture

To study the effect of the genetic variant in *TAF8* on the RNA and protein expression, primary fibroblast cell cultures were derived from skin biopsies taken from a TAF8-deficient fetus (VI14 Fig. 1A) and two normal control fibroblasts from healthy donors (not fetuses) using standard protocols. Fibroblasts were chosen since they are identical cell types with no differentiation potential. Fibroblast cells and nervous system cells both originate from ectoderm origin.

#### Western blotting

Proteins were extracted from fibroblasts of healthy donors and fetus VI14 as well as from embryos of WT mice. Western blot (WB) analysis was performed by using TAF8 primary antibody, Abcam (Waltham, MA, USA; Cat. No. ab204894) and anti-β-actin antibody, Cell Signaling Technology (Danvers, MA, USA; Cat. No. 8H10D10) at 1:250 concentrations. The secondary antibodies of the WB experiments were included IgG-HRP Goat Anti-Rabbit, Santa Cruz Biotechnology (Dallas, TX USA; Cat. No. SC 2004) and IgG-HRP Goat anti-Mouse, Invitrogen (Paisley, UK; Cat. No. A16072) at 1:2000 concentration. Membrane was filmed using G:BOX (Syngene, Frederick, WA, USA) with a chemiluminescence light.

#### cDNA synthesis

RNA and cDNA production was performed from fibroblast cells (affected VI14 and healthy controls) using standard protocol by qScript™ cDNA Synthesis Kit (Quanta Biosciences, Gaithersburg, MD, USA) according to the manufacturer’s instructions. cDNA amplicons for TAF8 and GAPDH were evaluated using multiplex analysis. GAPDH was used as a reference gene and normalized to healthy controls.

#### Sanger sequencing

Sanger sequencing was performed using ABI Prism 3500xl Genetic Analyzer, Applied Biosystems (Foster City, CA, USA). Standard protocols.

### Statistical analysis

Statistical analysis included *T*-test and one-way ANOVA test performed by Prism—GraphPad Software (GraphPad Software, Boston, MA, USA).

## Results

### Patients, pregnancy follow-up, and fetal neuro-sonography

Three couples from an extended Muslim Arab family (Fig. 1A) approached the genetics clinic as fetal brain malformations were discovered on fetal anatomy and neuro-sonography exams.

**Fig. 2:**
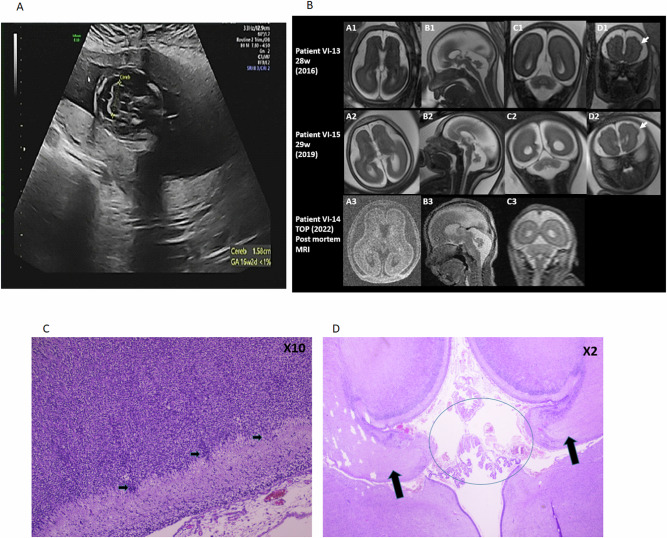
US, MRI, and pathological analysis of affected fetuses. **A** Ultrasound of brain at 19 weeks of VI13 demonstrating hypoplastic cerebellum (measurement between two calipers +) and enlarged cisterna magna. **B** MRI images of the three fetuses studied (VI13, VI14, VI15): axial T2 (A1, A2) and axial T1 (A3)—flat open sylvian fissure with small frontal lobes. Sagittal T2 (B1–B3)—dysmorphic corpus callosum (short and thick). Abnormal brain stem with flat pons. Abnormal small vermis, open fastigium, no primary fissure. Coronal T2—small TCD (C1–C3), abnormal gyral pattern (arrow) (D1, D2). **C** Affected TAF8 deficiency, fetal brain, showing leptomeningeal glioneuronal heterotopia (arrows), magnification—×10. **D** Demonstrating partial agenesis of the CC (circle) and intact parts of the CC (arrows), magnification—×2.

Pregnant woman #1 (V6, Fig. 1A) had fetal neuro-sonography at 24w + 5d which showed microcephaly, CC hypoplasia, and growth-restricted fetus. Delayed brain sulcation was observed, as was the fetus with constant bilateral clenched fists. Fetal brain MRI at 28 WG confirmed severe microcephaly and CC hypoplasia (VI13, Fig. 2B). The woman was referred for genetic counseling and subsequently underwent amniocentesis. The chromosomal microarray analysis yielded normal results. Ultimately, she opted for termination of pregnancy at 32 weeks resulting in the delivery of a male fetus weighing 1229 grams (5th percentile on local growth charts) [12]. During her fourth pregnancy, she again underwent pregnancy termination at 20 weeks due to the presence of similar fetal brain abnormalities (Fig. 2A) demonstrating cerebellar hypoplasia, confirmed on post-mortem brain MRI (VI14, Fig. 2B). She delivered a female fetus, birthweight 275 grams (<3rd percentile) (Table 1).

**Table 1:** Clinical diagnosis and follow-up in four fetuses.

Fetus no.	Week of diagnosis	Week of termination	Sonographic and MRI findings	Fetal weight at termination (percentile)
1	24w+ 5d	32w + 0d	• Microcephaly • Corpus callosum hypoplasia • Delayed brain sulcation	1229 g (5th)
2	19w + 0d	21w + 0d	• Cerebellar hypoplasia	275 g (<3rd)
3	23w + 2d	30w + 3d	• Short corpus callosum • Cerebellar hypoplasia • Microcephaly • Cortical atrophy • Delayed sulcation	1175 g (14th)
4	23w + 0d	31w + 0d	• Microcephaly • Severe brain atrophy	1141 g (9th)

Pregnant woman #2 (V8, Fig. 1A) underwent fetal neuro-sonography exam at 26w + 2d which revealed a short CC, cerebellar hypoplasia, and microcephaly. Fetal brain MRI was performed at 29 WG and revealed the above-mentioned findings (VI15, Fig. 2B). The pregnancy was terminated at parents’ request at 30w + 3d; a male fetus with birthweight of 1175 grams (14th percentile) was delivered (Table 1).

Pregnant woman #3 (VI5, Fig. 1A) underwent termination at 31w as fetal neuro-sonography confirmed microcephaly and severe brain atrophy. She underwent feticide and labor induction, and a growth-restricted female fetus of 1141 grams (9th percentile) birthweight was delivered (Table 1). A summary of the main clinical symptoms is provided in Table 1.

#### Brain MRI

Fetal MRI demonstrated additional findings including an abnormal sulcation pattern with open sylvian fissures, abnormal gyral pattern, small frontal lobes, abnormal brain stem with flat pons, and abnormal small cerebellum (Fig. 2B).

#### Molecular analyses

TAF8 deficiency was diagnosed in four affected fetuses (Fig. 1A). They were found to carry a homozygous pathogenic splicing variant, c.45 + 5 G > A, in *TAF8* which is a highly conserved splicing site (Ensembl 2024, Fig. 1B). The c.45 + 5 G > A in *TAF8* is a novel genetic variant (Clinvar SUB13973570, Fig. 1C); we determined it to be pathogenic in the four affected fetuses by segregation analysis within the nuclear families and biochemical analysis showing lack of TAF8 protein in an affected fetus. Population screening in the families’ village of residence revealed two carriers out of 77 individuals screened (2.6%), representing a founder genetic variant. There were no healthy individuals who were homozygous for this genetic variant.

#### Pathological analysis

Pathological evaluation of the female fetus: growth parameters were consistent with 20 WG, head circumference was 16 cm, microcephalic, (vs. 17.2 cm normal head circumference for 20 WG). No other external malformations were found. On internal inspection, the brain weight was 24 g (vs. 45.5 g normal weight for 20 WG). The brain appeared small for gestational age, the cortex appeared normal for gestational age. Partial agenesis of CC was noted (agenesis of the posterior part). Brain ventricles were not widened. Severe hypoplasia of the cerebellum was seen. Brain stem and pons were normal. Multiple sections of the cerebral hemispheres, cerebellum, and brain stem were performed.

**Fig. 3:**
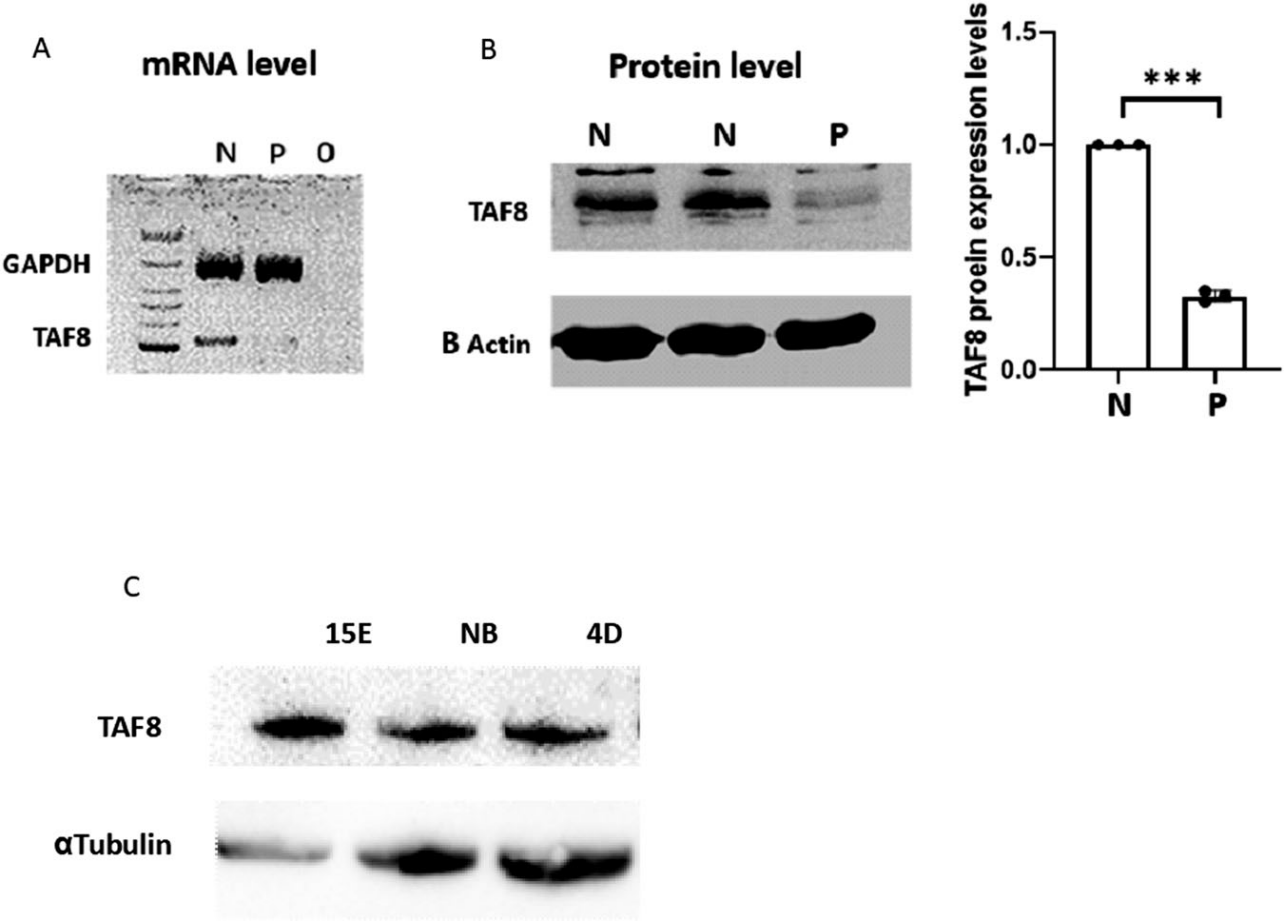
TAF8 protein and mRNA levels are reduced in an affected fetus. **A** mRNA multiplex analysis of *TAF8* in affected fetal (P) and normal control (N) fibroblasts. **B** WB analysis of TAF8 in affected fetal fibroblasts (P) and normal controls (N). ***p < 0.001 *T*-test, *n* = 3 in each group. **C** WB analysis of TAF8 at different developmental stages in mice. ***p < 0.001 one-way ANOVA test, *n* = 3 in each group.

On microscopic examination, abnormal development of the gyri and sulci with leptomeningeal glioneuronal heterotopia was seen (Fig. 3A, B), CC was thin, and cerebellar structure was abnormal with underdevelopment of the cerebellar hemispheres (Fig. 3C). Brain stem structure and the basal ganglia were normal. No other internal malformations were found.

#### Fibroblast cell culture analysis

To investigate the studied genetic variant effect on TAF8 mRNA and protein levels, proteins and RNA were extracted from fibroblasts derived from an affected fetus VI14 (P) and a healthy control (N). Significantly lower TAF8 protein expression and absence of TAF8 mRNA level were detected in P vs. N, *P* < 0.001 (Fig. 3A, B).

#### TAF8 expression in brain during the embryonic development

To study the importance of TAF8 during embryonic stages, the protein was extracted from the brains of WT mice at 4D (4 days), newborn (NB) and 15 embryonic days (15E) stages. WB analysis demonstrated significantly lower TAF8 expression at 4D (*p* < 0.001) and NB (*p* < 0.001) vs. 15E stage (Fig. 3C). Similarly, significantly reduced protein level of TAF8 was detected at 4D vs. NB (*p* < 0.001) (Fig. 3C).

## Discussion

We report, for the first time, fetal representation of TAF8 deficiency and document its importance in brain structural development.

TAF8 deficiency is a rare disease worldwide with only eight reported patients. We currently report four fetuses with a novel homozygous splicing genetic variant of *TAF8*, brain malformations, and restricted fetal growth (two fetuses from late-terminated pregnancies presented significant restriction in their growth while the other two were in the low normal range), meaning that TAF8 is a major player in normal brain development and might also affect growth patterns in the second and third trimesters.

Three of the fetuses underwent brain MRI (two prenatally and one post-termination at 20 WG) that supported the brain abnormalities already seen on US examination: flat open sylvian fissure with small frontal lobes, dysmorphic CC (short and thick), abnormal brain stem with flat pons, abnormal small vermis, open fastigium, hypoplastic cerebellum, and abnormal gyral pattern.

The brain anomalies that included abnormal sulcation patterns were previously described in TAF13 deficiency [8] and the progressive atrophy and abnormal CC seen on fetal MRI are like those described in patients with *TAF8* pathogenic genetic variants [1]. These brain abnormalities were proven on pathological examination of the last 20 WG fetuses that underwent brain autopsy, with very similar findings.

Although ultra-rare worldwide, we have identified three couples within the same small village where both parents are carriers of the same pathogenic genetic variant. The brain malformations seen in the homozygous fetuses, the lack of TAF8 determined in one affected fetus, and the segregation analysis in the extended family supports the pathogenicity of this novel genetic variant. To explore the possibility that affected sibs may carry additional loss of function (LOF) variants related to patients’ manifestations, exome sequencing studies were performed in two fetuses, at different laboratories. The results confirmed that at least two fetuses from the four affected had no other LOF genetic variant, nor were any other pathogenic/likely pathogenic variants identified. Moreover, in the context of a very homogenous pre-natal phenotype in all four fetuses, the same genetic variant was identified in all and the protein studies showed the loss of this important protein. One can safely say that this change in *TAF8* is causing the phenotype reported here.

Finally, we have identified a genetic island for TAF8 deficiency with a carrier frequency of 2.6% for this founder genetic variant, in this single village of Muslim Arab residents.

In Israel, this high carrier frequency justifies population screening followed by pre-natal or pre-gestational diagnosis for couples at risk of having a baby with this devastating disease.

Pre-natal diagnosis and counseling are crucial, as sonographic findings may be diagnosed late, in the second trimester. In all our cases, the brain was normal and adequate to fetal age in the early scan (around 15–16 WG). Cerebellar hypoplasia was the first clue to the development of this pathology, followed by CC dysgenesis and brain atrophy that were not detected before 19–20 WG. Our genetic findings enabled us to perform chorionic villus sampling in patient V6 in her present pregnancy, which revealed a non-homozygous fetus. Pregnancy is ongoing and will be followed carefully by neuro-sonography exams. The TAF8 mechanism of action on the developing brain is not yet known.

TFIID complex is a general transcription factor for RNA polymerase II. This complex contains several subunits such as TAF1, TAF2, TAF6, and TAF13 that were previously reported as causing monogenic neuro‐developmental disorders in humans [6]. In general, pathogenic variants in these genes as a group are associated with global developmental delay and dysmorphic features. Intellectual disability is the rule and all patients reported thus far were diagnosed postnatally [7, 8]. We now report, for the first time, pre-natal diagnosis for a TAF-related disorder caused by one of the crucial components in the TFIID complex—TAF8, known to affect the expression of many genes during the development of the central nervous system and crucial for the survival of neurons in the developing brain [13]. We have documented significant brain malformations in all four fetuses by fetal brain US including cerebellar hypoplasia, partial CC agenesis, and brain atrophy. Abnormal MRI findings were reported in other *TAF*-associated disorders and include mild-delayed myelination, discrete frontal pachygyria, and deep sulci of the cerebrum [8]. We hypothesize that the difference between the US imaging findings of those disorders and the TAF8 deficiency-affected fetuses and children might be due to the variable role of the TFIID subunits during the development of different brain regions [13–15].

Studies have also shown that TAF8 is important for mouse embryonic stem cell survival [10], evidence that might explain the phenomenon of restricted growth seen in the TAF8-deficient fetuses as well as the clinical manifestations which include post-natal growth retardation seen in patients with different *TAF-*associated disorders [6].

We studied the level of TAF8 protein expression in the whole brain of WT mice in embryonic and post-delivery stages and found that at embryonic stage 15E, TAF8 levels were significantly higher than in the NB and 4D stages. Our data support previous findings that showed a high level of TAF8 protein expression in the mice brain and mainly in the cortex at embryonic stages [13]. To the best of our knowledge, this is the first documentation in the literature for TAF8 expression patterns during different development stages of the brain showing that the main time of expression is in utero. Here we document that TAF8 has high expression levels in the brain of WT mice during the early embryonic development stage compared to the NB and early post-natal periods. These data support the significant role of TAF8 in early embryonic development of the brain.

Moreover, the four affected fetuses also presented moderate-to-severe restricted growth, suggesting that TAF8 is an important factor in fetal growth apart from its significance in brain development.

Our findings contribute to the understanding of TAF8’s role in the early development of the fetal brain and in fetal growth in general. Our report suggests that TAF8’s absence might affect the transcriptome and proteome. Furthermore, we show that pre-natal diagnosis for TFIID complex-related disorders is possible using fetal neuro-sonography techniques followed by exome sequencing, to diagnose severely affected fetuses with TAF-associated disorders as early as possible. The TAF subunit genes should be added to gene panels studied for brain anomalies. Finally, further investigation into TAF8’s function and mechanism of action could yield insights into their crucial biological and developmental roles in normal fetal development and possibly pave the way to potential therapeutic interventions for these devastating neurodevelopmental disorders.

## Data Availability

Additional data will be supplied upon reasonable request to the corresponding author. The details of the novel genetic variant reported here were disclosed to Clinvar. Clinvar SUB13973570.
